# What’s in a learning environment? Recognizing teachers’ roles in shaping a learning environment to support competency

**DOI:** 10.1007/s40037-015-0234-4

**Published:** 2015-11-02

**Authors:** Patricia S. O’Sullivan

**Affiliations:** University of California San Francisco School of Medicine, 533 Parnassus Avenue, Suite U80, Box 0710, 94143 San Francisco, CA USA

When educators in graduate medical education first focused on competency-based education, I remember a rush to find assessment instruments. I struggled to have those same educators pause long enough to think about how they would teach the competencies they planned to assess. Dijkstra and colleagues [1] in this issue have identified the importance of learning environment in preparing individuals who reported being ready to practice once they left residency. These results will encourage medical educators to include a focus on the learning environment when crafting educational programmes. While that is valuable, I want to raise awareness about how, through teaching, faculty can have a major effect on the learning environment.

The finding that learning environment affects achievement has appeared previously in higher education [2] and medical education [3]. In fact, the learning environment has risen to such significance that accrediting bodies have taken note. The Liaison Committee for Medical Education (LCME) [4] requires that schools conduct ‘periodic evaluation of the learning environment.’ The Accreditation Council for Graduate Medical Education uses site visits to assess the learning environment related to the institution’s efforts to engage residents in ‘patient safety; health care quality, including reduction in health care disparities; transitions in care; supervision; duty hours and fatigue management and mitigation; and professionalism’ [5]. This emphasis on learning environment has led to reviews of assessments that capture student and resident perceptions of the learning environment [6, 7] and ongoing efforts to develop new measures [8].

This rising interest made me wonder where teachers and teaching practice fit in the concept of a learning environment. Schronrock-Adema and colleagues [7], through their review of existing instruments, concluded that the domains of learning environment relate to goal orientation, relationships and organization/regulation. Dijkstra and colleagues [1] wrote statements assessing the learning environment from this framing. While teaching is not explicitly mentioned, the majority of statements used to evaluate the learning environment certainly related to teaching and to learner motivation. This is true for most learning environment instruments.

In my opinion, many items in learning environment instruments reflect self-determination theory (SDT). In brief, SDT, as put forth by Ryan and Deci [9] and summarized in medical education by Ten Cate [10], proposes that motivation is based on three needs: mastery, relatedness and autonomy. Mastery inspires learners to drive toward more challenging opportunities. Relatedness allows learners to feel connected and safe in an environment. Autonomy is expressed by an ability to initiate actions of one’s own volition. The Dijkstra items asked about examining performance and reflection (mastery), relationships and interactions with supervisors and peers (relatedness), and independence (autonomy). Teachers, in their role in the learning environment, must teach to support and actively incorporate elements of motivation.

Interestingly, Lemley et al. [11] examined the learning environment for the 21^st^ century student using the same lens of SDT. Lemley found that millennial students valued relevance of content, autonomy, having choices, and having connection with their teachers as emphasized by two-way conversations, respect, care, and knowledge of the student. Schumacher et al. [12] drew on SDT as part of their model to develop the master learner. The framing of learning environment around elements that support learner motivation is critical whether it relates to secondary school students, medical students, or resident learners. Those elements clearly come under the teaching mission.

Given the importance of creating a learning environment that motivates, the faculty member must establish this learning environment; and this is feasible because faculty have the opportunity to control how they teach. Medical educators in the clinical environment have proposed instructional strategies they believe align teaching with today’s millennial learner preferences [13]. Of the variety suggested, from ‘flipping the wards,’ to ‘embedding teaching moments into rounds,’ few capitalize on strategies related to motivation, particularly of the type suggested by self-determination theory.

Therefore, as teachers we must ensure that when using innovative and fun teaching strategies, we embed elements that motivate our learners to do better. So how might we do this? As we honour diverse teaching strategies, we need to redefine them to include steps that cannot be overlooked or forgotten. For mastery, we need frequent formative assessments in both didactic and clinical experiences. These can range from quizzes, to one-minute papers, to brief structured observations, or a patient note review. Assessments must lead to meaningful feedback that gives guidance on how to improve [14]. Additionally, time within teaching has to exist for reflection so that we allow learners to pause, think about how they are learning, and consider how they can engage in ways to improve their learning [15]. Setting aside this time and making it a natural part of teaching may be the most challenging of all. For relatedness we need to build in engagement with others. There is great value in learning from and with one’s peers [16] and patients [17]. For autonomy we need to include choices for learners, versus prescribing every way in which they will interact within a teaching method. This could be as small as where to sit. All faculty members must re-examine their teaching strategies to include these three elements. We will need faculty development to facilitate these changes.

Recently, we discovered that many of these teaching elements, such as steeping children in critical thinking and reflection, are already used in schooling prior to medical school. We hosted a session with local educators (some dealing with learners as young as 3 and 4 years old) entitled, ‘What will our health professions students of the future look like? Talk to those who teach them today!’ From their descriptions, we recognized that medical educators will need to make medical school classrooms and clinics welcoming to learners who already possess skills associated with self-determination theory. Additionally, these teachers told about extensive faculty development to learn these strategies; many teachers participate in professional learning communities to improve themselves as teachers and maximize learning for students [18]. These communities may be ideal to help medical educators to invigorate the learning environment.

In summary, the learning environment is critical in competency-based education. The way teachers teach has a major influence on the learning environment. We advocate that faculty redefine their teaching strategies to incorporate elements that reinforce motivation to enhance learning and that institutions provide them the support to do so.
